# Study on the Long-Term Salt Release Characteristics of Self-Melting Ice Asphalt Mixtures and Their Impact on Pavement Performance

**DOI:** 10.3390/polym16101379

**Published:** 2024-05-12

**Authors:** Chenyang Liu, Dedong Guo, Xupeng Sun, Xiang Li, Meng Xu, Massimo Losa, Chiara Riccardi, Teng Wang, Augusto Cannone Falchetto

**Affiliations:** 1 School of Transportation Civil Engineering, Shandong Jiaotong University, Jinan 250357, China; 22107025@stu.sdjtu.edu.cn (C.L.); 401002@sdjtu.edu.cn (D.G.); 21107010@stu.sdjtu.edu.cn (X.L.); 2 Department of Civil and Industrial Engineering, University of Pisa, Largo Lucio Lazzarino, 56122 Pisa, PI, Italy; losa@ing.unipi.it (M.L.); chiara.riccardi@unipi.it (C.R.); tw@chd.edu.cn (T.W.); 3 School of Highway, Chang’an University, South 2nd Ring Road Middle Section, Xi’an 710064, China; 4 Department of Civil Engineering, Aalto University, 02150 Espoo, Finland; augusto.cannonefalchetto@aalto.fi

**Keywords:** self-melting ice asphalt mixture, Los Angeles abrasion tester, long-term salt release patterns, pavement performance

## Abstract

Self-melting ice asphalt pavement materials inhibit pavement freezing and improve driving safety. This paper aims to study the long-term salt release characteristics of self-melting ice asphalt mixtures and the impact on pavement after complete salt release. Firstly, a method to accelerate the rapid release of salt based on the Los Angeles abrasion tester. Then, long-term salt release patterns were elucidated under the influence of deicing agent dosage, type of asphalt, and type of gradation. Finally, a quantitative analysis of the pavement performance after complete salt release is conducted. The results indicate that the release efficiency of the Los Angeles abrasion tester method has increased by 91 times compared to the magnetic stirrer immersion flushing method and by 114 times compared to the natural soaking method. The SBS-modified self-melting ice asphalt mixture possesses a longer duration of salt release, but the uniformity of salt release is inferior. Salt release duration is directly proportional to the dosage of deicing agents. SMA-13 self-melting ice asphalt mixture exhibits poorer uniformity in salt release. After complete salt release, high-temperature stability of self-melting ice asphalt mixtures decreased by 31.6%, low-temperature performance decreased by 15.4%, water stability decreased by 26.7%, and fatigue life decreased by 35.9%.

## 1. Introduction

The formation of ice on pavement diminishes the surface’s traction [1,2], significantly compromising pavement safety and transportation efficiency [3,4]. Addressing methods to reduce winter pavement icing has become a focal point of interest among numerous scholars [5,6]. Currently, pavement snow removal methods primarily involve manual snow removal [7], mechanical snow removal [8,9], and the application of deicing salt [10]. However, these methods all, to varying degrees, consume traffic resources and suffer from delayed deicing effects [11], thereby failing to address icy road surfaces promptly [12,13]. In this context, proactive snow melting and deicing technology for pavement has emerged as a crucial research subject.

Presently, proactive snow melting and deicing technologies primarily include self-stress elastic pavement technology [14,15,16], self-melting ice asphalt pavement [17], phase change material pavement [18,19,20], and energy conversion pavement [21,22,23]. Compared to the other three methods, self-melting ice asphalt pavement technology involves substituting deicing agents for aggregates or mineral powders during asphalt mixtures, which are mixed and laid down on the road surface. Under the influence of capillary force, osmotic pressure, and wheel “pumping,” solutes effectively diffuse from the confined spaces with higher internal concentrations in the mixture to the lower salt concentration surface of the road. As a result, this process disrupts the bond between ice and the road surface, lowering the freezing point of ice and snow on the road surface [24,25], thus restoring the pavement’s anti-skid functionality. Therefore, self-melting ice asphalt mixtures have sparked significant interest among researchers. This study focuses on the long-term salt release characteristics of self-melting ice asphalt mixtures and their impact on pavement performance.

To investigate the long-term salt release patterns of self-melting ice asphalt pavements, Zhong et al. [26] utilized a conductivity meter to measure the salt leaching amount of high-elasticity storage asphalt mixtures at various temperatures, studying the influence of temperature on the salt release patterns. Zheng et al. [27] analyzed the variation in salt release rate with temperature via the natural soaking method, obtaining the dissolution patterns of salt at different temperatures. Li et al. [28] researchers utilized the solution conductivity analysis method based on the principle of similarity to study the precipitation variation in low-freezing-point fillers in low-freezing-point asphalt mixtures, proposing a long-term prediction model. Yang Xiong [29] obtained the critical bonding force and critical concentration by analyzing the relationship between bond strength and effective components. Yu Yang [30] analyzed the deicing and snow-melting performance of low-freezing-point pavements by continuously measuring the release of chloride ions from soaked specimens. Wu et al. [31], using a Hamburg wheel-track device, simulated the effects of factors such as rainfall and load on salt dissolution. They established a salt precipitation prediction model with variables including anti-icing filler dosage, void ratio, rainfall intensity, temperature, and time. The addition of anti-freezing materials can have negative effects on the low-temperature cracking resistance and water stability of asphalt mixtures. Additionally, salt release can weaken the pavement performance of self-melting ice asphalt mixtures. Han et al. [32] utilized Mafilon (MFL) as a substitute for mineral powder to prepare self-melting ice asphalt mixtures. They found that pavement performance decreased with increasing MFL content. Using high-elasticity and high-viscosity asphalt binders [33,34,35] and fibers [36] can mitigate the negative impact of deicing agents on the pavement performance of asphalt mixtures. Zhang et al. [37] discovered via freeze–thaw tests that after 14 freeze–thaw cycles, the water stability, high-temperature stability, and low-temperature cracking resistance of self-melting ice asphalt mixtures decreased by 14.19%, 18.79%, and 11.96%, respectively. Zhang et al. [38] pointed out that selecting granular anti-freezing agents can alleviate the adverse effects of salt release on pavement performance. The impact of anti-freezing materials on the pavement performance of asphalt mixtures is mainly related to factors such as the dosage [39,40,41], type [42,43], application method [44], and gradation type [45] of deicing agents.

Currently, existing research on self-melting ice asphalt mixtures has several gaps:

- There is a lack of test methods to determine salt release patterns quickly.
- There is a lack of multifactor analysis, with the analysis of factors influencing salt release being relatively limited in scope.
- There is insufficient research on the pavement performance of self-melting ice asphalt mixtures after complete salt release.

Therefore, this study designs indoor experiments to accelerate rapid salt release, analyzing the long-term salt release characteristics of self-melting ice asphalt mixtures. It investigates the effects of different salt release methods, deicing agent dosages, asphalt types, and mixture gradation types on salt release. Furthermore, it analyzes the pavement performance of asphalt mixtures after complete salt release. The research findings hold important guiding significance for long-term evaluation methods of snow melting and deicing, as well as sustainable research on self-melting ice asphalt pavements.

## 2. Material and Experimental Design

### 2.1. Materials and Proportioning Design

#### 2.1.1. Asphalt and Aggregates

In accordance with the JTG E20-2011 [46], an examination was conducted on the Qilu brand 70# base asphalt and SBS-modified asphalt. As delineated in Table 1, the test outcomes manifest that the 70# base asphalt and SBS-modified asphalt performance metrics align with the technical requisites specified in the JTG F40-2004 [47].

The mineral aggregates used in this experiment underwent basic technical index testing in accordance with the requirements stipulated in the JTG E42-2005 [48]. The test results are detailed in Table 2 and Table 3. The experimental findings affirm that the properties of various aggregates and mineral powder gradations all conform to the specifications.

#### 2.1.2. Deicing Agent

**Figure 1:**
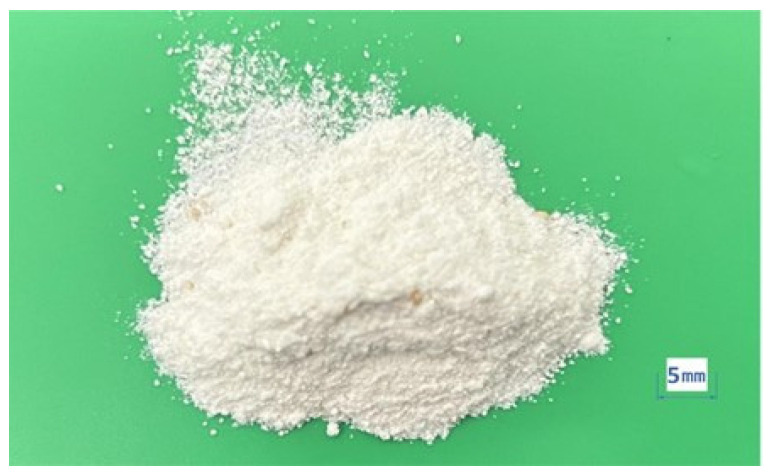
Deicing agent.

This study employed anti-freezing materials produced by a company in Henan, Figure 1 shows the appearance of the sample. By the specifications outlined in JT/T1210.2-2018 [49], the properties of the materials were tested. The test results are summarized in Table 4. 

#### 2.1.3. Standard Steel Hangers

This study employs standard steel hangers produced by a steel company in Shanxi Province. In accordance with the specifications outlined in GB/T 699-2015 [50] for Grade 20 steel, with dimensions of 50 × 25 × 5 mm^3^. Before conducting the experiment, remove the anti-corrosive oil from the surface of the standard steel hangers using filter paper. Immerse the specimens in anhydrous ethanol, followed by placing them in an oven for drying.

#### 2.1.4. Mix Proportion Design

In this experiment, considering the pavement performance of self-melting ice asphalt surfaces and the long-term release effectiveness of deicing agents in self-melting ice asphalt mixtures, as well as the influence of different structural types of self-melting ice asphalt mixtures on salt release, the study selected AC-13 dense-graded and SMA-13 stone mastic asphalt mixtures for gradation design based on the JTG E20-2011 [46] specification, with initial void ratios relatively small. The gradation data is presented in Table 5. Mineral aggregate gradation. The initial anti-freezing agent was added to the asphalt mixture by replacing 100% of the mineral powder in an equal volume manner. The optimal asphalt-to-aggregate ratio was determined using the Marshall mix proportion design method, resulting in ratios of 4.5% and 6.1%, respectively.

### 2.2. Experimental Design and Research

#### 2.2.1. Design of Salt Release Experiment Method

Salt release in self-melting ice asphalt mixtures is the process by which salt continuously precipitates from the surface or interior of the asphalt binder and dissolves in water to form a saline solution. Refer to relevant literature [51,52]. The three salt release experiment designs are as follows:

1. Utilizing the Los Angeles abrasion tester YDMH-A to abrasively scrub the self-melting ice asphalt mixture aggregates. Due to the disparity between gravity and barrel wall resistance, facilitating the process of mixing-separation-mixing between the self-melting ice asphalt mixture and water, achieving the repeated flushing effect of moisture on self-melting ice asphalt mixtures. This accelerates the release of salt from the asphalt surface or interior.
2. The molded specimens are placed into a magnetic stirrer, and the magnetic stirrer is utilized to induce the rotation of water flow, thereby facilitating the release of salt from the self-melting ice asphalt mixture.
3. Placing the self-melting ice asphalt mixture aggregates into a beaker and immersing them in water to promote the release of salt.

**Figure 2:**
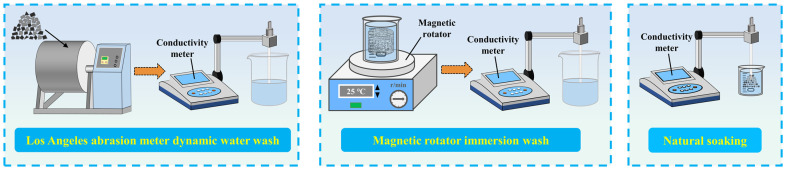
Experimental apparatus diagram.

The salt solutions obtained from the three experimental methods are all subjected to salt concentration measurements using the conductivity meter DDSJ-308F. Figure 2 shows the test device and process.

#### 2.2.2. Study of Parameters Influencing Salt Release Experiments

In this study, the added water’s magnitude determines the salt release rate. At the same time, the Los Angeles abrasion tester’s rotational speed and the magnetic stirrer dictate the water flushing frequency on the asphalt mixture within a given time frame. Drawing insights from relevant experimental research, taking the Los Angeles abrasion tester as an example, preparations were made for the dispersion of 70# base asphalt and AC-13 self-melting ice asphalt mixture aggregates. The deicing agent was introduced in a volumetric substitution of 100%, replacing mineral powder. The experimental design involves setting the rotation speed of the Los Angeles abrasion tester and the magnetic stirrer to a specific value denoted as ‘m’. Additionally, the mass ratio of the self-melting ice asphalt mixture to water for each of the three test methods is represented by ‘n’, as indicated in Table 6.

Utilizing the standard deviation of the concentration difference between adjacent unit time single salt releases during the salt release period as a measure, the uniformity of salt release is characterized. The calculation formula is given by Equation (1).

(1) $$U=\sqrt{\frac{{\sum_{i=2}^{T-1}\left(k_{i}-\bar{k}\right)}^{2}}{T-2}},$$

In the Formula (1), U represents the uniformity of salt release; $k_{i}$ denotes the slope of salt release concentration change between adjacent times; k represents the average slope; *T* is the salt release time, where T≥i≥2, about i is an integer.

#### 2.2.3. Research on Salt Release Patterns under the Influence of Multiple Factors

In this section, the authors analyze and study the salt release situation of self-melting ice asphalt mixtures under different influencing factors using the three test methods described in Section 2.2.1. 

Deicing agents were incorporated into the aggregates in volumetric substitutions of 100%, 60%, and 30%, respectively, replacing mineral powder. A 70# base asphalt was used to prepare the AC-13 asphalt mixture. The aggregates and the formed standard Marshall specimens underwent dynamic water flushing, immersion flushing, and natural soaking methods. The salt released was quantified, and the pattern of salt release was analyzed.

To investigate the influence of different types of asphalt on the salt release patterns in self-melting ice asphalt mixture, AC-13 self-melting ice asphalt mixture aggregates and standard Marshall specimens were prepared using 70# base asphalt and SBS-modified asphalt. The deicing agent was incorporated in a volumetric substitution of 100%, replacing mineral powder. Salt release experiments were conducted, revealing the salt release patterns for different types of asphalt.

In engineering applications, the three most widely used gradation types for asphalt mixtures are AC, SMA, and OGFC. The design void ratio of OGFC is generally greater than 18%, resulting in a higher void ratio. This leads to an excessive release of salt within a short period, making it challenging to control. In this section, AC-13 and SMA-13 self-melting ice asphalt mixtures were prepared using 70# base asphalt. To prevent the experimental variables, AC-13, with a different gradation, was mixed with a deicing agent in a volumetric substitution of 100%, replacing mineral powder. For SMA-13, the deicing agent quantity was equivalent to the mass of AC-13, with a volumetric substitution replacing mineral powder. The study explores the salt release under dynamic water flushing, immersion flushing, and natural soaking methods for different gradation types.

#### 2.2.4. Study on the Impact of Salt Permeation on Pavement Performance

##### High-Temperature Stability Test

To accurately reflect the impact of salt infiltration on the high-temperature stability of self-melting ice asphalt mixtures. Prepared specimens of 70# base asphalt and AC-13 type self-melting ice asphalt mixtures for groups A and B, with deicing agent contents of 0%, 30%, 60%, and 100%. For group B specimens, the deicing agent was completely released using the rotational scouring of the magnetic stirrer.

Group A and the released specimens from Group B were subjected to rutting tests with specimen dimensions of 300 × 300 × 50 mm^3^ following the specifications outlined in JTG E20-2011 T 0719 [46]. The test was conducted at 60 °C with a wheel pressure of 0.7 MPa. After 60 min, the dynamic stability was recorded. The calculation formula is given by Equation (2). The average values from the three sets of experiments were used to evaluate the high-temperature stability of self-melting ice asphalt mixtures with different deicing agent contents after complete release.

(2) $$DS=\frac{(t_{2}-t_{1})\times N}{d_{2}-d_{1}}\times C_{1}\times C_{2},$$

In Formula (2), DS represents the dynamic stability (cycles/mm), $d_{1}$ and $d_{2}$, respectively, correspond to the rut deformation at 45 min and 60 min, $C_{1}$ denotes the coefficient for the type of testing machine, $C_{2}$ represents the experimental coefficient, and N represents the testing wheel’s reciprocating rolling speed.

##### Low-Temperature Stability Test

**Figure 3:**
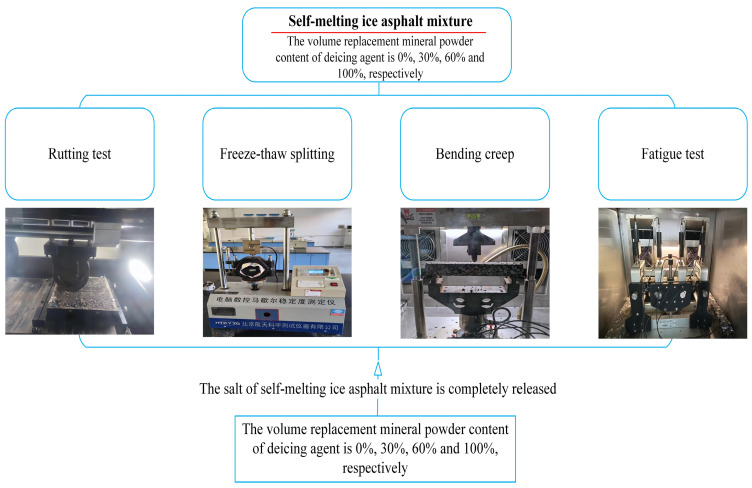
Pavement performance test graph.

Utilizing low-temperature bending beam tests to study the low-temperature shrinkage performance after the complete release of different deicing agent contents. The experiments were conducted according to the specifications outlined in JTG E20-2011 T 0715 [46]. Preparing specimens of 70# base asphalt and AC-13 type self-melting ice asphalt mixtures for groups C and D, with deicing agent contents of 0%, 30%, 60%, and 100%, for low-temperature bending beam tests. For group D, low-temperature bending beam specimens were subjected to rotational scouring using the magnetic stirrer to release the deicing agent completely. The specimen dimensions were 250 × 30 × 35 mm^3^. The specimens were loaded using a three-point bending method with a span of 200 mm until fracture occurred, at a loading rate of 50 mm/min. The test was conducted at −10 °C, as shown in Figure 3. The calculation formula is given by Equation (3).

(3) $$S_{B}=\frac{L^{3}P_{B}}{4bdh^{3}},$$

In the Formula (3), $S_{B}$ denotes the bending stiffness modulus, and $P_{B}$ denotes the maximum load at specimen failure.

##### Water Stability Test

The freeze–thaw splitting test can simulate the long-term effects of moisture on self-melting ice asphalt mixtures in a short time. According to the specifications outlined in JTG E20-2011 T 0729 [46]. Preparing specimens for groups E and F with deicing agent contents of 0%, 30%, 60%, and 100%. These specimens were subjected to double-sided compaction 50 times to create Marshall specimens. For group F, the Marshall specimens were subjected to rotational scouring using the magnetic stirrer to release the deicing agent completely. Subsequently, groups E and F were each divided into two further groups, denoted as G, H, I, and J, respectively.

Groups G and H were stored at room temperature for standby. Groups I and J underwent vacuum saturation for 15 min at a vacuum level of 97.3–98.7 kPa. After returning to atmospheric pressure, they were left at room temperature for 0.5 h before being subjected to a freezing treatment at −18 °C for 16 h. The specimens were immediately transferred to a constant-temperature water bath at 60 °C for 24 h. Subsequently, all groups (G, H, I, and J) were immersed in a water bath at room temperature (25 °C) for 2 h before measuring their splitting strength. The calculation formula is given by Equation (4).

(4) $$TSR=\frac{{\bar{R}}_{T2}}{{\bar{R}}_{T1}}\times 100,$$

In the Formula (4), TSR denotes the strength ratio in freeze–thaw splitting tests, ${\bar{R}}_{T2}$ denotes the average tensile splitting strength after freeze–thaw cycling, and ${\bar{R}}_{T1}$ denotes the average tensile splitting strength before freeze–thaw cycling.

##### Four-Point Bending Fatigue Life Test

To accurately portray the change in fatigue life following the complete release of salt content from the self-melting ice asphalt mixture. According to specification JTG E20-2011 T 0739 [46], specimens of 70# base asphalt and AC-13 self-melting ice asphalt mixture with deicing agent contents of 0%, 30%, 60%, and 100% for groups M and N were prepared. The dimensions of the specimens are 380 × 50 × 63 mm^3^. For group N specimens, the deicing agent was completely released using the rotational scouring of the magnetic stirrer. 

The specimens from groups M and N were placed in an environmental chamber maintained at a test temperature of ±0.5 °C for 4 h for conditioning. The displacement sensor was adjusted with its pulley in contact with the surface of the specimen and then positioned at the midpoint of the specimen. Under the target test strain level, preloading of 50 cycles was conducted, and the specimen’s modulus of elasticity at the 50th loading cycle was calculated to be equal to the initial modulus of elasticity. Once the initial modulus is determined, the four-point bending fatigue testing machine automatically adjusts and stabilizes to the target tensile strain level required for the test while simultaneously monitoring and recording the test results. Automatically cease loading when the specimens reach the termination criteria of the fatigue test. The calculation formula is given by Equation (5).

(5) $$S=\frac{12\delta LP}{\omega h(3L^{2}-4\alpha^{2})},$$

In the Formula (5), S denotes the bending stiffness modulus, δ denotes the maximum strain at the center of the beam, L denotes the beam span, P denotes the peak load, ω denotes the width of the beam, h denotes the height of the beam, α denotes the distance between the centers of adjacent clamps.

#### 2.2.5. Study of the Influence of Salt Precipitation Extract on Steel

**Figure 4:**
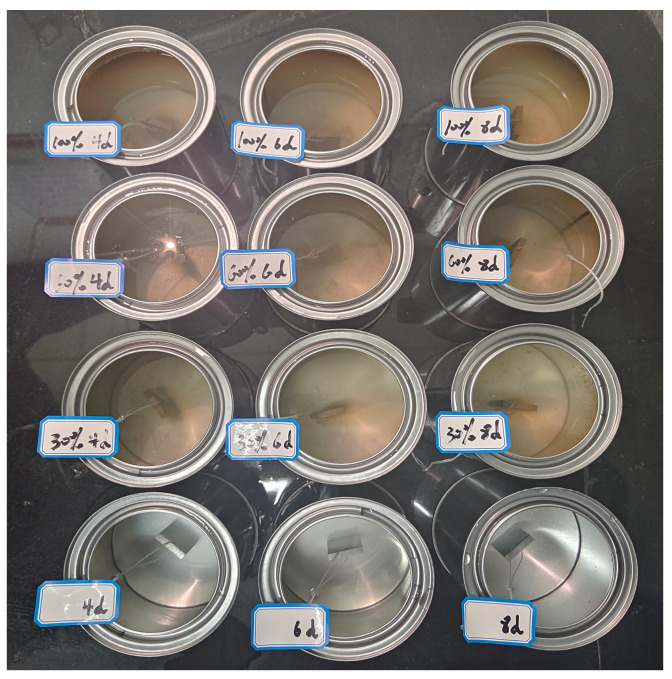
Corrosion testing of standard steel hangers.

Based on the change in mass of the standard steel hangers before and after corrosion. Assess the influence of salt precipitation extract on steel corrosion. Figure 4 shows a standard steel hanger placed in a salt precipitate. Label the standard steel hangers as Group X, Group Y, Group Z, and Control Group, respectively. Three specimens per group. Immerse t the standard steel hangers labeled as Group X, Group Y, and Group Z completely in the precipitation solution released from the self-melting ice asphalt mixture with deicing agent concentrations of 30%, 60%, and 100%, respectively, for 12 h. Then, suspend the standard steel hangers in the air for 12 h. Group X undergoes 4 cycles, equivalent to 4 days; Group Y undergoes 6 cycles, equivalent to 6 days; Group Z undergoes 8 cycles, equivalent to 8 days. The control group is treated with deionized water.

After the cycling period, immerse the rusted hanger specimens in a rust removal solution. After cleaning, place the specimens in an oven for drying. Use an analytical balance with a precision of 0.1 mg to accurately measure the mass of each of the standard steel hangers and record the mass loss. It should be noted that the concentration of the salt precipitation extract obtained from the experiment is much higher than that of the salt precipitation extract from actual self-melting ice asphalt pavement. The corrosion of steel is much more severe under experimental conditions compared to a real-world environment.

## 3. Results and Discussions

### 3.1. Salt Release Test Method Analysis

**Figure 5:**
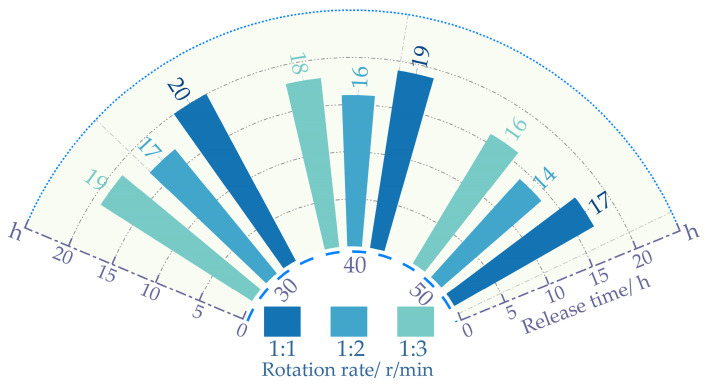
Rotation rate and release time.

**Figure 6:**
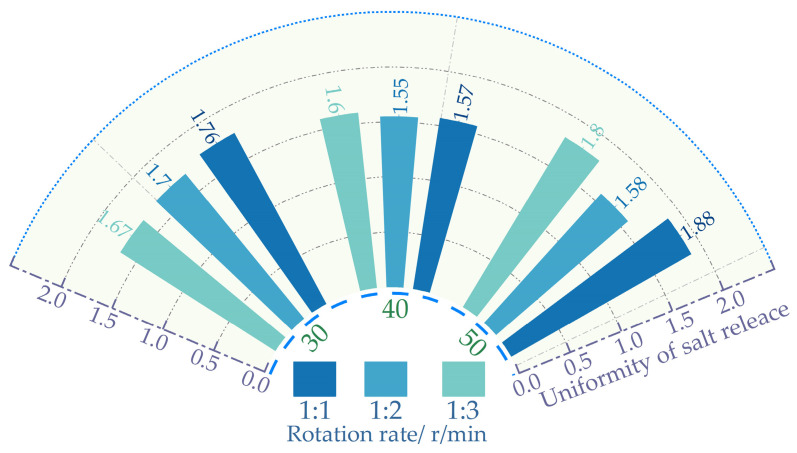
Rotation rate and uniformity of salt release.

Figure 5 shows that at the same proportion, the salt release time decreases as the rotation speed increases. With the amplification of the rotational velocity, the frequency of water impacting the mixture intensifies. Accelerating the release of salt content within the asphalt. This results in a reduction in the release time for salt content in self-melting ice asphalt mixtures. Excessive rotational speed may lead to premature detachment of asphalt from the aggregate surface. The asphalt, once detached, sinks into the water, with salt release occurring at an extremely sluggish pace. Resulting in diminished uniformity in salt release. From Figure 6, it is evident that when the rotation speed is 40 r/min, the process of mixing, separation, and re-mixing of the mixture with water is fully achieved, resulting in the best uniformity. At the same rotational speed, Excessive water content can also lead to premature detachment of asphalt from the surface of the mixture. Insufficient water content impedes the complete realization of the mixing–separation–mixing process between the mixture and water. In summary, a series of experiments have determined that the optimal process parameters for both the Los Angeles abrasion tester and the magnetic stirrer involve a rotational speed of 40 revolutions per minute (rpm). The water-to-mixture mass ratio for all three experimental methods is 1:2.

### 3.2. Analysis of Regulatory Patterns in Salt Release Affected by Various Factors

Utilizing the methods of the Los Angeles abrasion tester, magnetic stirrer, and natural soaking, this study investigates the impact of varying deicing agent concentrations, asphalt types, and asphalt mixture structures on salt release. 

In the figure, showed that 70# base asphalt, AC-13 type asphalt mixture, with deicing agent contents of 100%, 60%, and 30%; SBS-modified asphalt, AC-13 type asphalt mixture, with a deicing agent content of 100% and 70# base asphalt, SMA-13 type asphalt mixture, with a deicing agent content of 100%.

**Figure 7:**
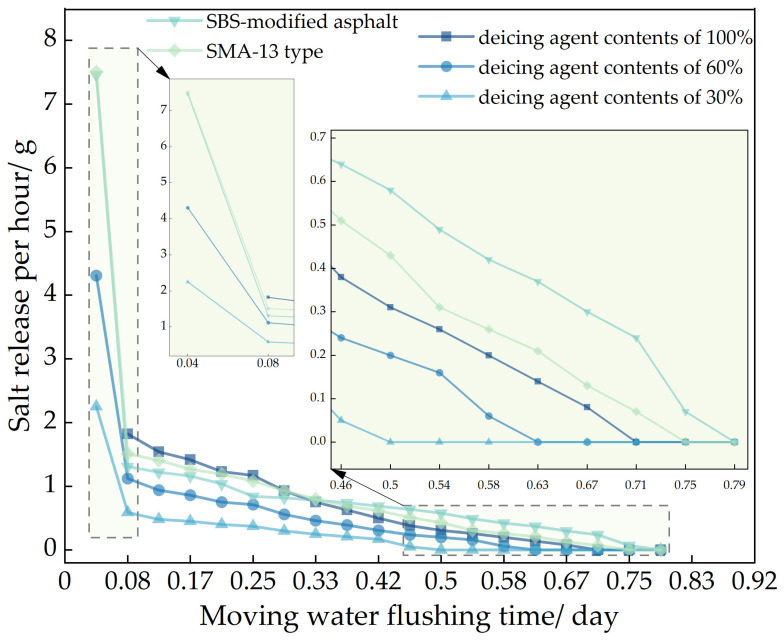
Salt release data from dynamic water flushing.

**Figure 8:**
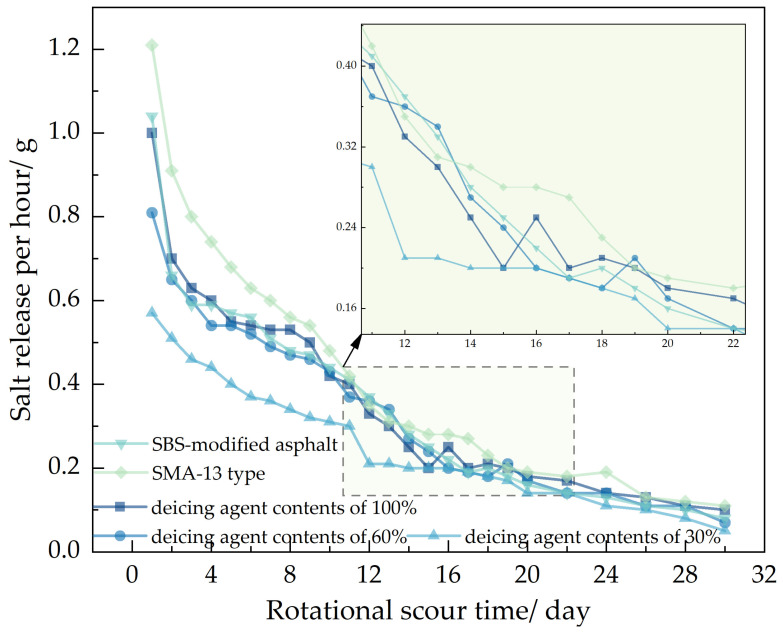
Soaking and flushing salt release data.

**Figure 9:**
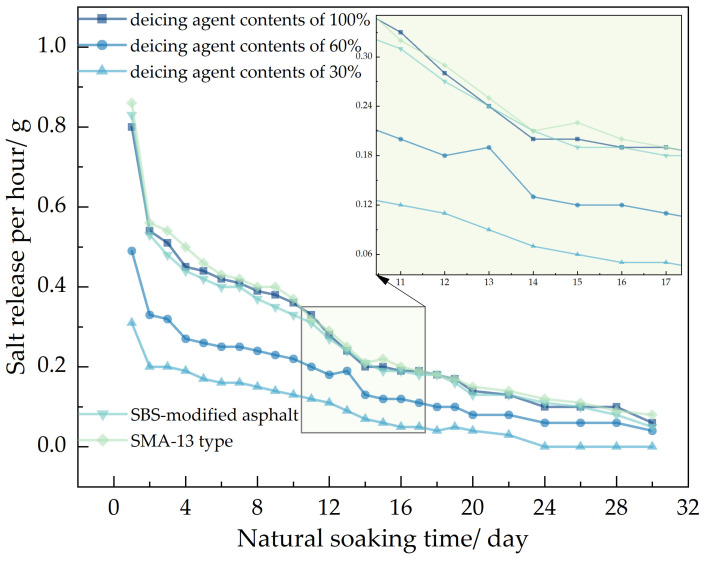
Salt release data from natural soaking.

It can be seen from Figure 7, Figure 8 and Figure 9, using the Los Angeles abrasion tester to accelerate the salt release of self-melting ice asphalt mixtures, the salt release rate increased by 91 times compared to the magnetic stirrer immersion flushing method and by 114 times compared to the natural soaking method. Analyze the reasons for this phenomenon, due to the high rotational speed of the Los Angeles abrasion tester driving the water flow, resulting in a strong flushing force exerted by the water flow on the self-melting ice asphalt mixture. Salt is continuously released from the surface or interior of the asphalt emulsion.

In the analysis of salt release trends across the three testing methods, it was observed that self-melting ice asphalt mixtures using SBS-modified asphalt exhibited a longer salt release duration compared to those using 70# base asphalt. However, SBS-modified asphalt mixtures showed poorer uniformity in salt release. Analyze the reasons for this phenomenon. Deicing agents primarily exist within the asphalt emulsion, while SBS-modified asphalt is a three-dimensional structure formed by adding an SBS modifier to the base asphalt and then subjecting it to shear and mixing. Some deicing agents are placed within a mesh-like structure, constrained by the mesh-like structure, the release process of salt is more challenging, salt cannot be fully released, and the release time is prolonged, resulting in poor uniformity of salt release.

Regarding different deicing agent contents in self-melting ice asphalt mixtures, the salt release time was directly proportional to the deicing agent content, with minimal impact on the uniformity of salt release. When comparing asphalt types and deicing agent contents, it was found that, under similar conditions, SMA-13 asphalt mixtures exhibited poorer uniformity in salt release compared to AC-13 asphalt mixtures. Analyze the reasons for this phenomenon. SMA-type asphalt mixtures belong to a skeletal-dense structure, characterized by a high proportion of coarse aggregates, mineral filler, and asphalt, with fewer fine aggregates; compared to AC-type asphalt mixtures, SMA-type asphalt mixtures have a large flushing area, the excessive area causes the mixture to be initially affected by moisture, resulting in an excessive release of salt, resulting in poor uniformity of release.

### 3.3. Analysis of the Impact of Salt Seepage on Pavement Performance

#### 3.3.1. Analysis of High-Temperature Stability

**Figure 10:**
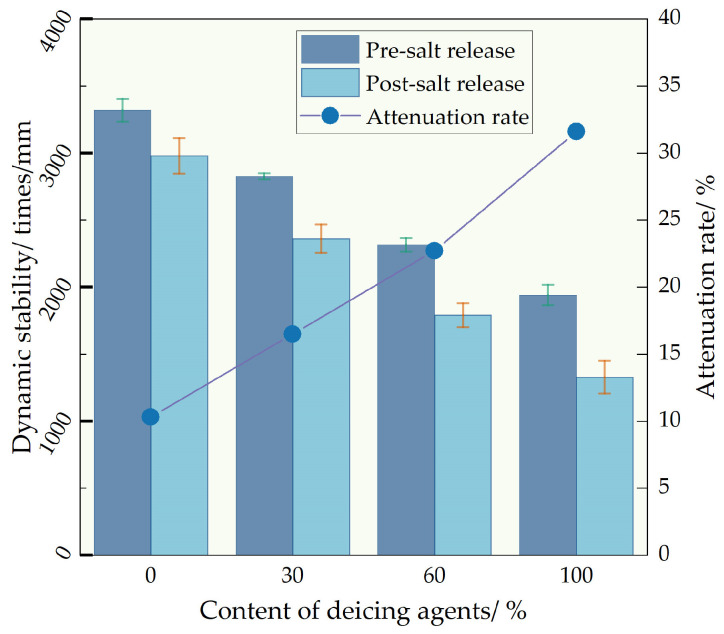
High-temperature stability test results comparison.

The impact of deicing agent concentrations on the high-temperature performance variation in self-melting ice asphalt mixture after complete salt release was investigated via trajectory testing. It can be seen from Figure 10. With an increase in deicing agent concentration, the high-temperature performance of the self-melting ice asphalt mixture experiences a decline. Before salt release, the dynamic stability of deicing agent concentrations D30, D60, and D100 decreased by 14.9%, 30.2%, and 41.5%, respectively, compared to the D0 concentration. This decline can be attributed to the less effective interaction between the deicing agent and asphalt compared to the interaction between alkaline limestone filler and asphalt. Consequently, the adhesion between asphalt mortar and aggregate is compromised, leading to a reduction in the dynamic stability of the mixture.

Salt release adversely affects the high-temperature performance of self-melting ice asphalt mixture, and an increase in the blending ratio exacerbates the degradation of high-temperature performance. After salt release, the self-dynamic stability of D0, D30, D60, and D100 concentrations decreases by 10.3%, 16.5%, 22.7%, and 31.6%, respectively. The emergence of porous silica pores after salt release increases the specimen’s porosity. Simultaneously, salt precipitation alters the properties of the asphalt binder, resulting in a decline in adhesion between asphalt and aggregate, thereby reducing the high-temperature resistance to rutting of the asphalt pavement.

#### 3.3.2. Analysis of Low-Temperature Stability

**Figure 11:**
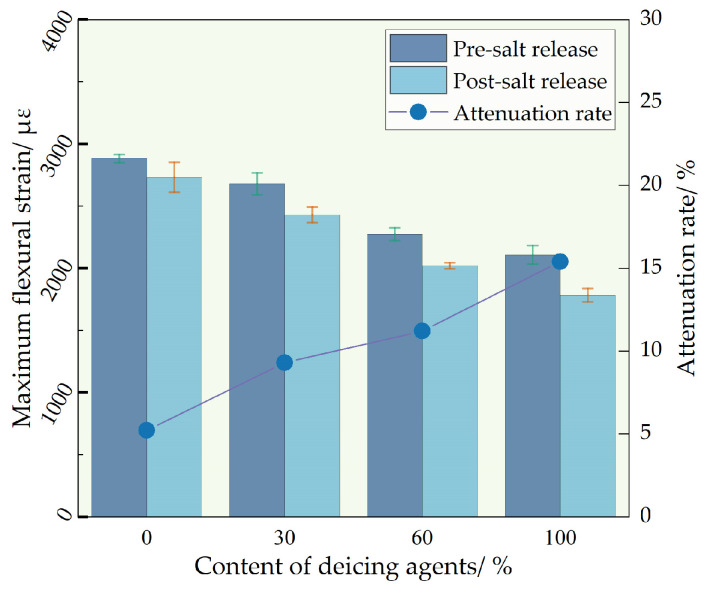
Low-temperature stability test results comparison.

The variations in the low-temperature performance of self-melting ice asphalt mixtures before and after salt release were investigated via beam flexural testing and can be seen in Figure 11. The addition of deicing agents adversely affected the low-temperature performance of self-melting ice asphalt mixtures, with a more significant decrease observed as the anti-freezing agent dosage increased. Prior to salt release, the maximum bending strain of D30, D60, and D100, relative to D0, decreased by 7.1%, 21.1%, and 26.9%, respectively. This decline is primarily attributed to the weakening of the interaction between aggregates and asphalt due to the addition of deicing agents, reducing low-temperature performance. Additionally, deicing agents are primarily distributed in the asphalt, and their dispersal characteristics may reduce the continuity of asphalt on the fracture surface, diminishing its toughness and consequently affecting the low-temperature performance of the mixture. After complete salt release, the maximum bending strain of self-melting ice asphalt mixtures is somewhat reduced, with a significantly greater decrease than conventional asphalt mixtures. Following salt release, the maximum bending strain of D0, D30, D60, and D100 concentrations decreases by 5.2%, 9.3%, 11.2%, and 15.4%, respectively. This reduction may be attributed to the release of deicing agents after immersion, causing partial damage to the internal structure of the asphalt mixture. Furthermore, the porosity increases with the release of chloride ions in the mixture, further disrupting the adhesive relationship between asphalt and aggregate and weakening crack resistance performance.

#### 3.3.3. Analysis of Water Stability

**Figure 12:**
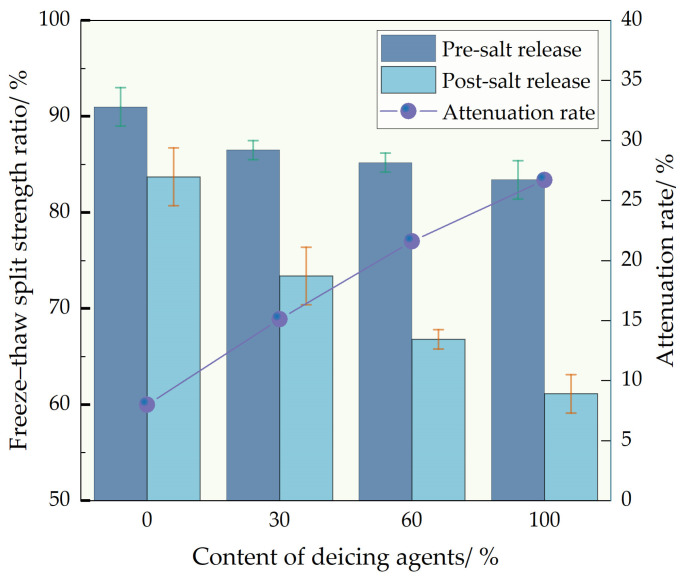
Water stability test results comparison.

Based on the variation in the freeze–thaw splitting strength of the self-melting ice asphalt mixture with the release of salt, an analysis was conducted on the influence of deicing agent dosage and salt precipitation on the water stability performance of the asphalt mixture. The experimental results are depicted in Figure 12. It is evident that with the increase in deicing agent dosage, the splitting strength significantly decreases, indicating an adverse impact of the deicing agent on the water stability of the mixture. Before salt release, the freeze–thaw splitting strength ratios of D30, D60, and D100 to D0 decreased by 4.9%, 6.4%, and 8.4%, respectively. This is attributed to the deicing agent being unfavorable for moisture resistance, as its increased dosage leads to a reduction in structural asphalt within the asphalt, consequently compromising the integrity of the asphalt mixture and causing a decline in moisture resistance performance.

Furthermore, after the deicing agent dissolves in water, the micro-pores of the asphalt mixture enlarge, making it easier for water to penetrate the interior of the asphalt mixture. This could be one of the reasons for the decrease in the residual stability ratio. With the complete release of salt, the freeze–thaw splitting strength of all specimens significantly decreases, with a more pronounced decrease in the freeze–thaw splitting strength of asphalt mixture specimens with an added deicing agent. After salt release, the maximum bending strain of D0, D30, D60, and D100 concentrations decreases by 8%, 15.1%, 21.6%, and 26.7%, respectively. A plausible explanation is that the deicing agent continues to release underwater immersion conditions, and chloride salt solution erodes the interface between asphalt and aggregate. Simultaneously, the permeation of chloride ions leads to the dissolution of the light components of asphalt, accelerating the aging of asphalt binder and further reducing the adhesive properties between asphalt and aggregate. This results in asphalt being more prone to detach from the aggregate surface, leading to a deterioration in the water stability performance of the mixture.

#### 3.3.4. Analysis of Four-Point Bending Fatigue Life Test

**Figure 13:**
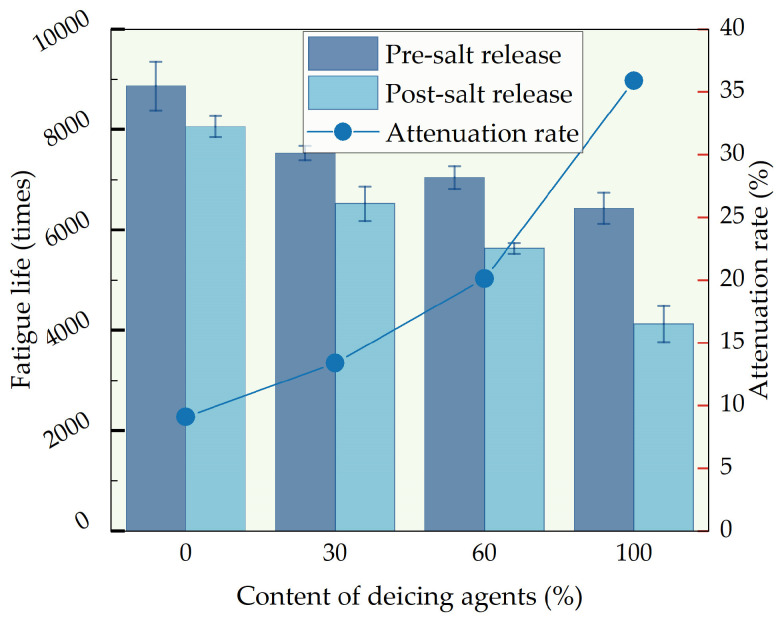
Four-point bending fatigue life test results comparison.

Using a four-point bending fatigue testing machine to determine the fatigue life of compacted asphalt mixtures under repeated bending loads. From Figure 13, it is evident that prior to salt release, the fatigue life of D30, D60, and D100 decreased by 15.1%, 20.5%, and 20.75%, respectively, compared to D0. The addition of de-icing agents is demonstrated to diminish the fatigue resistance of asphalt mixtures. This is due to the infiltration of chloride ions, leading to a decrease in the content of lightweight components in asphalt, resulting in the hardening and brittleness of the asphalt mixture. Therefore, under repeated loading, fatigue failure is more likely to occur, resulting in a lower fatigue life being manifested.

After complete salt release, the fatigue life of D0, D30, D60, and D100, respectively, decreased by 9.1%, 13.4%, 20.1%, and 35.9%. This phenomenon arises from the ease with which cations such as sodium and magnesium in the salt solution interact with the polar components in asphalt, forming organic metal salts with high solubility. The organic metal salts erode the adhesive interface between asphalt and aggregates, significantly weakening the adhesive strength formed between the aggregates and asphalt. This results in the asphalt on the surface of the mixture detaching from the aggregate surface, further reducing the fatigue life of the self-melting ice asphalt mixture.

### 3.4. Analysis of the Influence of Salt Precipitation Extract on Steel

**Figure 14:**
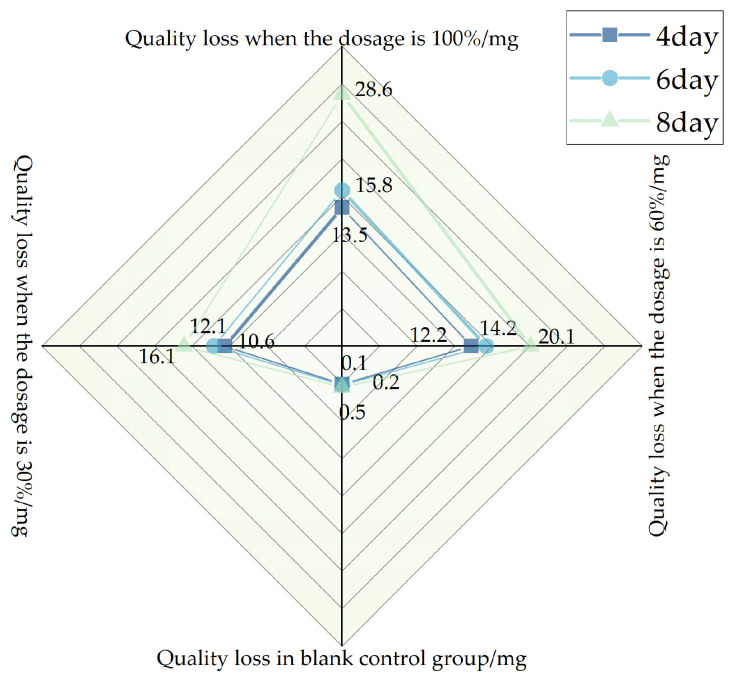
Loss of weight from corrosion of standard steel hangers.

The investigation focuses on the corrosion of standard steel hangers caused by leachates with varying salt concentrations. Based on the results of the corrosion test depicted in Figure 14. The saline solution used for precipitation accelerates the corrosion of the standard steel hangers. Under equivalent immersion durations, when the concentration of the deicing agent is at 100%, the corrosion of standard steel hangers is most severe. By the eighth day, the corrosion rate can reach 0.06%. Through the analysis of the reasons, As the concentration of salt precipitates increases, the rate of electrochemical reaction of standard steel hangers is accelerated. As a result, the corrosion of standard steel hangers has deepened. The experimental design considers the most adverse conditions. The actual concentration of salt in the runoff from self-melting ice asphalt pavement is significantly lower than the concentration of precipitated solution in the experiment. Thus, the corrosion rate of steel caused by the salt analysis runoff from self-melting ice asphalt pavement should be less than 0.06%. Hence, in actual environmental conditions, the impact of self-melting ice asphalt mixture on the corrosion rate of steel is minor.

## 4. Conclusions

This paper uses the Los Angeles wear meter to accelerate the salt release of self-melting ice asphalt mixture and obtains the long-term salt release rules of different anti-icing agent dosages, asphalt type, and gradation type from the conventional test piece immersion flushing method and natural soaking method and evaluates the pavement performance of self-melting ice asphalt mixture after the complete release of salt. The conclusion is as follows:

- Based on the Los Angeles abrasion tester, dynamic water flushing was conducted on the aggregates of self-melting ice asphalt mixtures too thoroughly mix, separate, and re-mix the asphalt mixture with water. The optimal process parameters were determined to be a rotation speed of 40 r/min and a water-to-mixture mass ratio of 1:2.
- Accelerating the release of salt from self-melting ice asphalt mixtures was achieved using the Los Angeles abrasion tester. Compared to conventional tests using the magnetic stirrer immersion flushing method, the efficiency was increased by 91 times, and compared to the natural soaking method, it was increased by 114 times.
- Self-melting ice asphalt mixtures using SBS-modified asphalt exhibit longer salt release durations, demonstrating poorer uniformity. The salt release time is directly proportional to the dosage of the deicing agent. The dosage of the deicing agent has minimal effect on the uniformity of salt release. When the type and dosage of asphalt are consistent, SMA-13 asphalt mixtures exhibit poorer salt release uniformity than AC-13 asphalt mixtures.
- As the proportion of deicing agents replacing mineral powder increases, the performance of self-melting ice asphalt mixtures gradually deteriorates. After the complete release of the salt, the pavement performance tends to deteriorate compared to its original state. When replacing the mineral powder with a 100% deicing agent, the high-temperature stability of the self-melting ice asphalt mixture decreased by 31.6%, the low-temperature performance decreased by 15.4%, the water stability decreased by 26.7%, and the fatigue life, respectively, decreased by 35.9% after the complete salt release.
- Under laboratory conditions, when the deicing agent entirely substitutes the mineral powder in the asphalt mixture, The standard steel hangers immersed in the fully precipitated saline solution for 8 days exhibited a weight loss rate of 0.06%. Due to the significantly higher salt concentration in the extracted solution compared to that generated by the self-melting ice asphalt pavement in actual environmental conditions, The corrosion impact on steel materials in actual road surface environments is relatively minor.

The indoor experimental methods mentioned can accelerate the release of salt content. The salt release characteristics observed in indoor experiments differ from those encountered in using self-melting ice asphalt pavements.

## 5. Future Research

The research on self-melting ice asphalt mixtures should also investigate how deicing agents are released in a more uniform manner. For instance, whether they can be incorporated into slurry sea. Additionally, existing deicing agents contain a significant amount of chlorine elements. Studying environmentally friendly self-melting ice asphalt mixtures is highly necessary.

## Figures and Tables

**Table 1:** The 70# Base asphalt, SBS-modified asphalt technical property index.

Test Items	Asphalt Kinds	Test Results	Technical Requirements
Penetration (25 °C, 100 g, 5 s) (0.1 mm)	70# base asphalt	70.8	60~80
	SBS-modified asphalt	52.5	40~60
Ductility (5 cm/min)/cm	70# base asphalt	>100 (15 °C)	≥100
	SBS-modified asphalt	58.8 (5 °C)	≥50
Softening point (ring and ball method) (°C)	70# base asphalt	47.3	>46
	SBS-modified asphalt	78.4	≥60
Power viscosity (Pa·s)	70# base asphalt	215 (60 °C)	≥180
	SBS-modified asphalt	1.5 (135 °C)	≤3

**Table 2:** Indicators of technical properties of mineral powder.

Test Items	Test Results	Technical Requirements
Water content (%)	0.42	≤1
Hydrophilicity coefficient	0.70	<1
Plasticity index (%)	2.5	<4

**Table 3:** Indicators of the technical properties of aggregates.

Test Items		Test Results	Technical Requirements
	Particle size (mm)	-	-
Apparent relative density	9.5~16.0	2.777	≥2.45
	4.75~9.5	2.794	
	0~2.36	2.754	
Water absorption (%)	9.5~16.0	0.65	≤3.0
	4.75~9.5	0.78	
Sand equivalent (%)	0~2.36	77.8	≥60
Crushing value (%)		21	≤30
Wear value (%)		12.4	≤35
Needle flake content (%)		5.67	≤15
Adhesion		Level 4	≥Level 4

**Table 4:** Basic indicators for deicing agent.

Test Items	Chloride Content (%)	Heat Resistance Index (%)	Relative Density	Nominal Maximum Particle Size (mm)	Water Content (%)
Test results	58.9	0.30	2.25	0.075	0.7
Technical requirements	≥35	≤0.5	≥1.7	≤0.3	≤1

**Table 5:** Mineral aggregate gradation.

Sieve size (mm)		16	13.2	9.5	4.75	2.36	1.18	0.6	0.3	0.15	0.075
Passing rate (%)	SMA-13	100	95.9	64.8	27.6	21.3	18.1	16.2	14.7	13.2	11.5
	AC-13	100	95.8	75.3	44.9	29.7	21.5	14.0	10.4	8.9	6.0

**Table 6:** Values of the factors in the accelerated salt precipitation test.

Serial Number	Rotation Rate (m)/(r/min)	Mix: Water Addition (n)
1	30	3:1
2	40	2:1
3	50	1:1

## Data Availability

The experimental data in this paper are from the pavement material laboratory of Shandong Jiaotong University, which is the provincial key laboratory.
